# ARTIFICIAL INTELLIGENCE IN THE HISTOLOGICAL DIAGNOSIS OF COLON CANCER: A RAY OF HOPE

**DOI:** 10.1590/S0004-2803.24612025-095

**Published:** 2025-12-01

**Authors:** Akhilesh Vikram SINGH, Anudwipa SINGH, K. Vanitha PRAKASH

**Affiliations:** 1 Graphic Era Deemed to be a University, Dehradun, Uttarakhand, India.; 2 Sri Sai Jyothi College of Pharmacy, Hyderabad, Telangana, India.

Dear Editor,

Colon cancer remains a major cause of cancer-related deaths globally. It highlights the significance of early and accurate diagnosis in improving patient outcomes. Histopathological analysis, being the gold standard for colon cancer detection, is time-consuming, susceptible to interobserver variability, and necessitates the availability of efficient pathologists. The field of medical diagnostics has undergone significant changes following the advent of artificial intelligence (AI), particularly with the emergence of deep learning models, such as convolutional neural networks (CNNs). This new tool improved the accuracy, speed, and consistency in the diagnostic prospect. It has been documented that AI has transformed clinical and histopathological diagnosis by enhancing the process of cancer detection, segmentation, and classification across several imaging modalities, including histopathology, computed tomography (CT), magnetic resonance imaging (MRI), and positron emission tomography (PET), etc.^1^. Deep learning models like CNNs have attained high diagnostic accuracy, outperforming expert pathologists in the identification of colorectal cancer. Wang et al. (2021)^2^ developed deep learning-based AI models for the diagnosis of colorectal cancer with the help of histopathological whole-slide images. This model surpassed the diagnostic capability of professional pathologists, achieving an AUC of 0.988 after being trained on more than 170,000 image patches derived from 9,600 patients. These AI-generated heatmaps reduced the workload and diagnostic variability while increasing the accuracy rate of cancer diagnosis. This study thus demonstrated the promising role of AI-based technology and proved to be a trustworthy clinical tool that could be further modified for diagnosing various malignancies. These approaches have integrated various machine learning techniques, including noise reduction, feature extraction, and classification, utilizing multiple models. Hence, the performance is further enhanced, and high accuracy is achieved by using optimization techniques, which further facilitates early cancer detection^3^.

In **c**olorectal cancer, mismatch repair (dMMR) deficiency results in increased mutation rates and microsatellite instability (MSI). These are crucial for immunotherapy and Lynch syndrome screening. Conventional MSI testing techniques are often expensive and inaccessible. Thus, CNNs have proven to exhibit promising results in such cases wherein the transformative role of AI has been successful in colon cancer diagnosis, decreasing the workload, improving accuracy, and ensuring early detection with better patient outcomes. The integration of histological and genomic data to predict immunotherapy response, especially in the identification of microsatellite stable (MSS) tumors with MSI-like characteristics, has expanded the range of available treatments^4^. Tharwat et al. (2022)^5^ has emphasized the advances in AI-driven colon cancer diagnoses by focusing on deep learning models like AlexNet, VGG16, VGG19, ResNet50, DenseNet121, and InceptionV3 in polyp detection and cancer classification. Although these models have improved the specificity, sensitivity and accuracy, there have been complications like staining variability and limited annotated datasets. Another study by Hadiyoso et al. (2023)^6^, proposed a CNN-based model utilizing VGG16 architecture and Contrast Limited Adaptive Histogram Equalization (CLAHE) for classifying histopathological images of lung and colon cancer. Twenty-five thousand images were trained in this model and an accuracy percentage of 98.96 was attained. This represented a superior performance of CLAHE that enhanced the image contrast and classification accuracy.

Studies have shown a higher accuracy rate for customized CNN models compared to pre-trained networks, including ShuffleNet V2, GoogLeNet, and ResNet18, when evaluating their efficacies. It was evident that ShuffleNet V2 demonstrated remarkable efficacy in colon cancer detection with minimal training time. Hence, these findings suggest that the effectiveness of CNN-based models in medical diagnostics highlights the necessity of ongoing deep learning model modification to improve clinical applicability, reduce processing time, and achieve good accuracy^7^. Xiao et al. (2023)^8^ used tumor segmentation and quantitative morphological feature analysis to create a deep learning-based predictive model for colorectal cancer (CRC). The study used the Eff-UNet model to analyze the tumor-specific areas exclusively by precisely segmenting tumor regions from histopathology images. CellProfiler was used to extract features, while the Lasso-Cox model was employed for analysis. Cross-validation and Kaplan-Meier estimates were utilized to confirm the prognostic performance. These characteristics were associated with important cancer pathways, including immune escape, leukocyte adhesion, proliferation, and immune receptor activation, as determined by Gene Ontology (GO) enrichment analysis. The clinical relevance of this model was enhanced by its predictable accuracy, which was comparable to that of the TNM staging system. By focusing on tumor regions, this method enhanced biological interpretability, reduced variability from non-tumor areas, and improved statistical significance. Hence, this proved its potential for improved patient stratification and customized colorectal prognosis. The model performed better and showed more accuracy than non-segmentation-based methods.

A deep learning framework for predicting chromosomal instability (CIN) in colorectal cancer (CRC) was developed by Hyeon et al. (2024)^9^ using whole slide images. Tumors were categorized into groups with high (AS-H) and low (AS-L) aneuploidy scores based on nuclear morphology and copy-number signatures. ResNet18-SSL performed remarkably well in tile-based predictions, and CIN was associated with nuclear characteristics. A lightweight deep learning model (mobile device optimized) was proposed by Shahadat et al. (2024)^10^ for identifying colon cancer. The authors attained 100% accuracy on the LC25000 dataset. This tool further enhanced accessibility and enabled faster and more precise cancer diagnosis, resulting in improved patient outcomes.

As AI-driven models continue to evolve, their integration into medical practice could transform histopathological workflows and augment the expertise of histopathologists. These AI-based models have the capability to transform precision oncology with advancing computing power and improved imaging modalities. Thus, this would lead to a more accurate prognosis and modified treatment options for patients with colon cancer. AI in colon cancer histopathology has certain limitations despite its accuracy. AI has limitations owing to the variability in staining methods, slide preparation, and imaging scanners. The absence of multi-center validation and uniform imaging restricts reliability, whereas small biopsies may not represent tumor heterogeneity in metastasis. A significant drawback is the deficiency of interpretability in deep learning models, thus obscuring clinical decision-making. Hence, future research should focus on developing nucleus-specific models and multimodal imaging using AI. Standardized protocols and diverse datasets are essential for reproducibility, while prospective clinical trials are necessary to validate the role of AI in CIN detection and personalized treatment.

## Data Availability

data usage not reported, research data not used.
